# Preservation of erniettomorph fossils in clay-rich siliciclastic deposits from the Ediacaran Wood Canyon Formation, Nevada

**DOI:** 10.1098/rsfs.2020.0012

**Published:** 2020-06-12

**Authors:** J. G. Hall, E. F. Smith, N. Tamura, S. C. Fakra, T. Bosak

**Affiliations:** 1 Department of Earth, Atmospheric, and Planetary Sciences, Massachusetts Institute of Technology, Cambridge, USA; 2 Department of Earth and Planetary Sciences, Johns Hopkins University, Baltimore, USA; 3 Advanced Light Source, Lawrence Berkeley National Laboratory, Berkeley, USA

**Keywords:** Ediacaran, fossil, three-dimensional preservation, taphonomy, clay minerals, X-ray spectroscopy

## Abstract

Three-dimensionally preserved Ediacaran fossils occur globally within sandstone beds. Sandy siliciclastic deposits of the Ediacaran Wood Canyon Formation (WCF) in the Montgomery Mountains, Nevada, contain two fossil morphologies with similar shapes and sizes: one exhibits mm-scale ridges and a distinct lower boundary and the other is devoid of these diagnostic features. We interpret these as taphomorphs of erniettomorphs, soft-bodied organisms with uncertain taxonomic affinities. We explore the cast-and-mould preservation of both taphomorphs by petrography, Raman spectroscopy, X-ray fluorescence microprobe and X-ray diffraction. All fossils and the surrounding sedimentary matrix contain quartz grains, iron-rich chlorite and muscovite. The ridged fossils contain about 70% larger quartz grains compared to the ridgeless taphomorph, indicating a lower abundance of clay minerals in the ridged fossil. Chlorite and muscovite likely originated from smectite and kaolinite precursors that underwent lower greenschist facies metamorphism. Kaolinite and smectite are inferred to have been abundant in sediments around the ridged fossil, which enabled the preservation of a continuous, distinct, clay- and kerogen-rich bottom boundary. The prevalence of quartz in the ridged fossils of the WCF and in erniettomorphs from other localities also suggests a role for this mineral in three-dimensional preservation of erniettomorphs in sandstone and siltstone deposits.

## 1. Background

Three-dimensional cast-and-mould preservation of soft-bodied organisms occurs rarely in the fossil record, but is a common taphonomic mode during the Ediacaran and lower Palaeozoic ([1] and references therein). Because exceptionally preserved fossils from this time may include deeply diverging animals, understanding the fossilization mechanisms during the Ediacaran and early Phanerozoic has garnered much attention. These fossils, commonly preserved in shale and siltstone deposits, have inspired numerous hypotheses about fossilization mechanisms such as pyritization [2–4], silicification (e.g. [5]) and aluminosilicification (e.g. [6,7]). In these taphonomic windows, pyrite, silica and aluminosilicates are thought to replace the original organic material and protect some organic molecules from decay (e.g. [4–6,8]). Three-dimensional cast-and-mould preservation—a taphonomic mode that may have preserved diverging metazoan clades during the Ediacaran [9,10]—is comparatively less well understood than other taphonomic windows. This mode of preservation has been hypothesized to require pyrite (e.g. [11,12]), silica [1] and/or aluminosilicates (e.g. [13–15]) to cement the mould and/or act as the moulding surfaces. If better understood, this style of preservation can tell us about the surrounding environmental conditions and the original tissue types, i.e. factors used to assess the lifestyles, diversity of taxa and types of organisms during the Ediacaran and early Palaeozoic [16–18]. Specifically, determining how different taphonomic windows preserve diagnostic characters directly informs interpretations of fossils [18–21].

The three-dimensional morphology of soft-bodied organisms can be preserved when the decay of soft tissues is delayed and precipitated minerals replace the tissues [8,22–24]. Chemical and physical conditions during the early stages of decay can influence mineral precipitation and delay the decay of soft tissue. Anoxic conditions, which are known to occur around unburied [22] and buried [23] decaying tissues, may limit microbial activity and the decay of organic material [22]. The abundance and composition of clay minerals within the sediment may additionally delay decay [25] if the adsorbed or authigenic clay minerals protect organic matter from degradation [6,26,27]. Taphonomy experiments have compared the roles of quartz, calcite, kaolinite and illite in delaying the decay of arthropods and other invertebrates [25,28–30], but it is not known how mineral mixtures that are common in natural sediments preserve different tissues. A recent study reported the formation of clay veneers around muscle tissue buried in kaolinite, demonstrating that authigenic clay minerals can form during delayed decay and preserve some features of soft-bodied organisms in clay-rich sediments [28]. Illite, kaolinite and their metamorphic products are often associated with Cambrian fossil assemblages [31,32] and are sometimes present in Ediacaran fossil assemblages [13,33,34]. It is debated whether these minerals were present during the initial fossilization process or formed later during metamorphism (e.g. [6,13,32,34–36]). Microbial reduction of iron and sulfate coupled to organic decay is thought by some to have enabled the precipitation of sulfide minerals such as pyrite around Ediacaran fossils found in sandstone beds [7,8,37]. However, others have suggested that iron phases associated with these beds formed during later diagenesis and not during the initial decay of the organism [38]. Pyrite has been directly observed in some fossil specimens (e.g. [4,13]), but some interpret iron oxides present within Ediacaran fossils as the result of pyrite oxidation (e.g. [11,12,39,40]). A recent study supported the relationship between sulfide minerals and porous sandstones by showing that greigite, an iron sulfide mineral that forms in acidic environments, and iron oxides can precipitate on muscle tissues buried in quartz sand [28]. Overall, the current uncertainties highlight the need to consider both early and late diagenetic pathways and metamorphism in fossil preservation.

**Figure 1.**
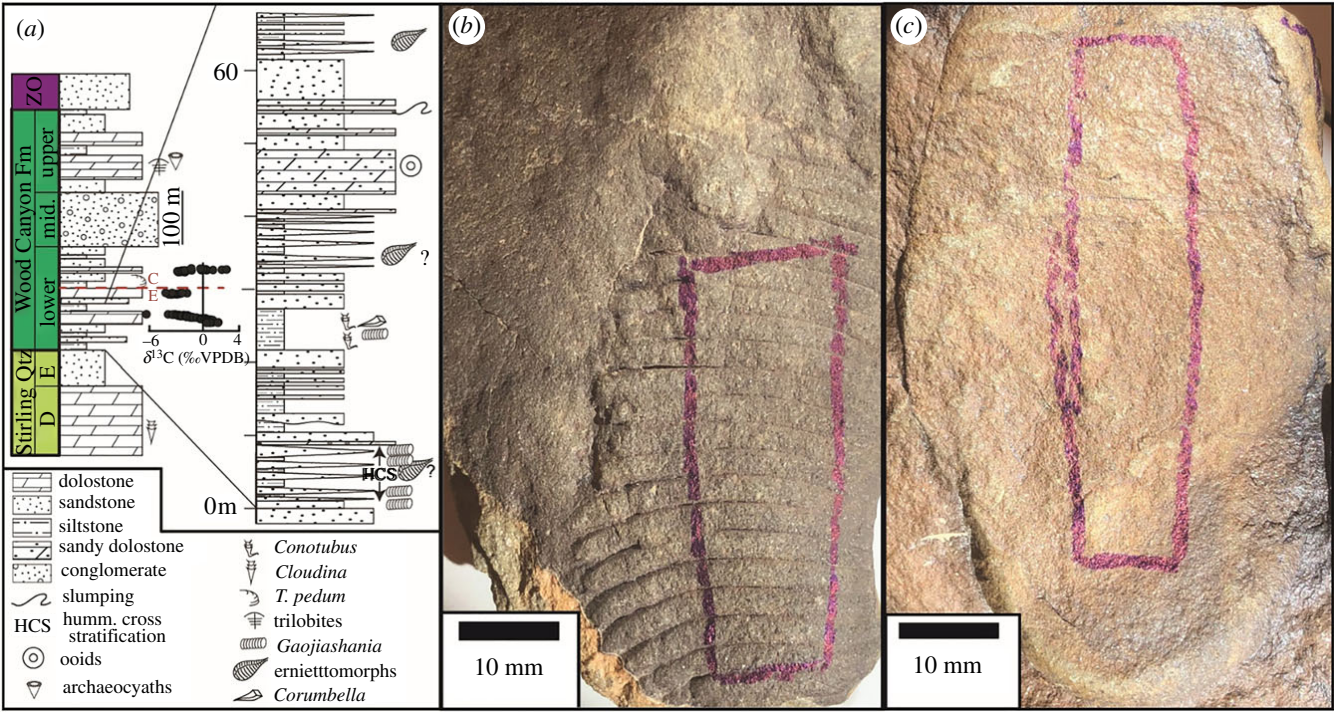
(*a*) Stratigraphic section of the Montgomery Mountains, Nevada area from Smith *et al*. [39]. (*b*) Erniettomorph fossil with mm-scale ridges. (*c*) Ridgeless erniettomorph fossil. The boxed regions show the lines along which the fossils were cut for thin sectioning and analyses.

Latest Ediacaran cast-and-mould-style and pyritized fossil assemblages have been discovered in the late Ediacaran to early Cambrian lower Wood Canyon Formation (WCF) in the Montgomery Mountains, Nevada [39,41–43]. Muscovite and chlorite clay minerals are present throughout the lower WCF, indicating that this formation underwent lower greenschist facies metamorphism [44]. Cast-and-mould-style and pyritized fossils are present in the same formation and often in the same types of sedimentary strata [39]. This offers an opportunity to examine preservation processes by analysing the distributions of minerals and carbonaceous material in the fossils and the surrounding sedimentary rock. Two distinct cast-and-mould-style oval erniettomorphs are reported in the micaceous sandstone beds of the WCF: one with mm-scale ridges and distinct lower boundaries and one with the identical oval outline, but flattened and without the boundaries and ridges (figure 1) [39]. The ridgeless specimens were tentatively identified as a taphomorph of an erniettomorph due to their uniform shapes and sizes and similarity to the ridged specimens [39]. The same study also reported abiotic sedimentary structures in the lower member of the WCF that could be difficult to distinguish from poorly preserved fossils. However, these authors tentatively assigned a biotic origin of the ridgeless structures because multiple specimens within one slab exhibited similar shapes and sizes and were also similar to the ridged erniettomorphs [39]. As such, we refer to these as ‘ridgeless erniettomorphs', but acknowledge that definitively assigning these to the morphoclade of erniettomorphs is difficult. Other ridged structures from the correlative Deep Springs Formation are superficially similar to tubular Ediacaran body fossils [45] and have actually been mistaken for erniettomorphs [46]. These ribbed and sheeted problematic structures occur in a different sedimentary facies, are interpreted as microbial in origin [45] and do not resemble either the ridged or the ridgeless erniettomoprhs either in shape or in size.

Here we seek to reconstruct fossilization processes, microbe–mineral interactions and conditions that preserved the macroscopic shapes and the finer features in the WCF erniettomorphs and address the origin of differences between the two taphomorphs. The mapped distributions of clay, oxide and sulfide minerals and organic matter within and around the fossils are used to assess the potential roles of allochthonous clay minerals and authigenic mineral phases in the early stages of fossilization.

## 2. Material and methods

### 2.1. Materials

Erniettomorph fossils were found within the lower WCF in the Montgomery Mountains, NV, and were previously described by Smith *et al*. [39]. Two ridged and four ridgeless structures were present in the respective original slabs, indicating that multiple fossils from individual slabs/beds exhibited the same quality and style of preservation. The fossil slabs and the fossils analysed here are shown in fig. 3*d*,*i* of the previous study [39]. One representative sample of each erniettomorph taphomorph was analysed in this study, and both are pictured in figure 1*b*,*c*. These specimens are reposited at the Smithsonian Institution National Museum of Natural History as catalogue numbers 642 300 (ridged) and 642 302 (ridgeless).

### 2.2. Methods

#### 2.2.1. Thin sections and petrography

The fossils analysed were cut with a Struers Labotom-5 rock saw along the boxes indicated in figure 1. One thin section of each fossil was prepared at Spectrum Petrographics (Vancouver, WA, USA). These thin sections were first viewed in transmitted and reflected light, under 2.5 × , 10 × and 20 × magnifications, using a Zeiss Axioscope A1 microscope equipped with a Zeiss AxioCam ERC 5 s and ZEN imaging software. Scales in petrographic images were inserted using ImageJ (v1.52a) and any noteworthy features were annotated in Adobe Illustrator (vCS5). The photomosaics were created from transmitted light ­­images taken at 2.5 × magnification in Adobe Illustrator. Three polished cross sections of each analysed fossil were made. These sections were viewed with an AmScope stereo microscope equipped with an Am 3MP digital camera and AmScope imaging software. The scale bars were added and the images were annotated in Adobe Illustrator.

Fifteen frames from the photomosaics of each thin section were chosen randomly and used for grain size analyses in ImageJ. Three 93 000 µm^2^ areas were selected in each of the randomly selected frames. A colour threshold was optimized for each frame to mark only the quartz grains and applied to the selected areas to measure the grain sizes and per cent area occupied by the quartz grains. Each selected area was also assigned a placement in the thin section: *f* for areas inside the fossil, *I* for areas at the interface and *o* for areas outside the fossil. The abundances of quartz grains in these regions were compared using independent t-test and an analysis of variance (ANOVA) using the NumPy and SciPy.stats packages in Python 3. We conducted a Levene's test using the SciPy.stats package before an ANOVA test to ensure that the data had equal variances. In cases where the ANOVA showed a statistically significant difference, a Tukey HSD *post hoc* analysis, which simultaneously compares means among all groups, was performed to find which regions had significantly different per cent area of quartz from each other. This test was done with the statsmodels.stats.multicomp package in Python 3.

#### 2.2.2. Energy-dispersive X-ray spectroscopy

The thin sections were analysed with the Supra 55VP FESEM at the Harvard University Center for Nanoscale Systems (CNS). Energy-dispersive X-ray spectroscopy (EDS) was performed on the areas that are marked by filled rectangles in the photomosaics shown in figure 3 using a working distance of 8.5 mm and a beam energy of 12 keV. Both spectra and maps were collected with the program EDAX Genesis (v6.54) available at the same facility. Line profiles of the EDS map images were made in ImageJ (v1.52a).

#### 2.2.3. X-ray diffraction

The ridged erniettomorph thin section was mounted onto a XY stage with double-sided tape and analysed by the Bruker D8 Gadds Multipurpose Diffractometer at the Center for Materials Science and Engineering (CMSE) at MIT. The spectra were collected as a line scan indicated by the red-dashed line in figure 3*a*. Each spectrum was collected for 8 min from 2*θ* values of 5 to 80 degrees over four frames using a glancing incident X-ray detector. The orientation of the sample relative to the X-ray beam was optimized to reduce the scan area along the width of the fossil. This was done to differentiate between areas along the line scan of the fossil. The data were then imported into the Bruker Diffrac Eva software and converted into a one-dimensional scan. These one-dimensional scans were subsequently analysed using the HighScore Plus software commercially available from Malvern Panalytical.

#### 2.2.4. X-ray microprobe

Thin sections were analysed using the X-ray fluorescence microprobe (micro X-ray fluorescence (µXRF), micro X-ray absorption spectroscopy (µXAS) and micro X-ray diffraction (µXRD)) at beamline 10.3.2 of the Advanced Light Source (ALS) at the Lawrence Berkeley National Laboratory (LBNL), Berkeley, CA [47]. X-ray fluorescence maps were collected at 7210 eV using a beam spot size of 7 × 7 µm, 20 × 20 µm pixels and a dwell time of 50 ms pixel^−1^. Micro-XRF spectra were also recorded on each pixel of the maps. Fe K-edge X-ray absorption near-edge structure (XANES) spectra were collected at the spots marked by filled circles in the photomosaic of the ridged fossil (figure 3*a*) and by empty yellow circles in the µXRF maps (figure 5*a,b*). Spectra were collected in quick-XAS mode where the Si(111) monochromator is scanned on the fly from 100 eV below to up to 300 eV above the Fe K-edge (7110.75 eV [48]). All data were collected in fluorescence mode using a 7elements Ge solid-state detector (Canberra, ON). Spectra were deadtime corrected, deglitched and calibrated using an Fe foil (first derivative taken at 7110.75 eV [48]). Spectra were then least-square linear combination fitted using a large XAS database of Fe bearing compounds and methods described elsewhere [28]. This method enables the identification of mineral groups (silicates, oxides, sulfides, sulfates, carbonates, native halides and phosphates) and in some cases mineral identity, depending on the robustness of the Fe-bearing standard XAS database. Hence, we consider mineral assignments obtained by this method as tentative. Additionally, Fe valence plots were generated from the Fe XANES spectra using normalized absorption values at 7113 eV and 7117.5 eV producing a two-dimensional scatter plot, following methods described elsewhere [47,49].

#### 2.2.5. Micro X-ray diffraction

The thin sections were analysed at the ALS µXRD beamline 12.3.2 at the LBNL, CA. Thin sections analysed by µXRD were taped onto the XYZ stage and analysed at a beam energy of 8 keV. Each frame was collected for 30 s using a DECTRIS Pilatus 1 M detector. Regions mapped in this manner are indicated by dashed rectangles in the photomosaic (figure 3). The spectra of individual frames from each region were added using the XMAS software and integrated from 2*θ* values of 13 to 65 degrees over chi values of −30 to 30 to create a one-dimensional scan. These scans were analysed in HighScore Plus to identify mineral phases.

#### 2.2.6. Raman spectroscopy

Minerals, organic matter and epoxy in thin sections were examined by the Hyperspectral Darkfield Raman microscope located in the CNS facility at Harvard. Excitation laser at 405 nm was used and the Raman shifts were collected from 50 to 2200 cm^−1^ with a grating of 2400 gr mm^−1^ or from 150 to 3200 cm^−1^ with a grating of 1800 gr mm^−1^. The spectra and maps were collected over 5–10 s intervals with 100% of the laser energy. The slit size was 100 µm and the hole size was 300 µm. Spectra analysed using the KnowItAll software were background fitted and subtracted in the LabSpec 6 software commercially available from Horiba Scientific. The spectra were also analysed with a generated MATLAB (vR2019a) code based on methods described in McNeil *et al*. [50]. This method takes the ratio of a background-subtracted spectrum and the maximum value reached in the fluorescence band, defined as the range greater than 1700 cm^−1^. Another MATLAB (vR2019a) code was generated to determine the maximum temperature attained by carbonaceous material, using the equation described in Beyssac *et al*. [51].

## 3. Results

### 3.1. Fossil morphology and appearance in hand samples

The erniettomorph fossils were found in pieces of float from the sandstone and siltstone beds (figure 1). They stood out from the surrounding sediments in the float pieces, likely due to differential weathering along the fossil surfaces. The upper surfaces of both fossil types and exterior sediments weathered to a darker colour relative to the interior sediments. Two morphotypes were evident in two different hand samples. Both were approximately 6 cm long and 3.5 cm wide, with similar ovoid shapes. Millimetre-scale ridges and a possible suture line were apparent on the top surface of one morphotype, the other had no diagnostic features present. The ridgeless fossil appeared flattened compared to the ridged fossils, as noted by Smith *et al*. [39]. Bottom surfaces of the fossils were not apparent in the hand samples.

**Figure 2.**
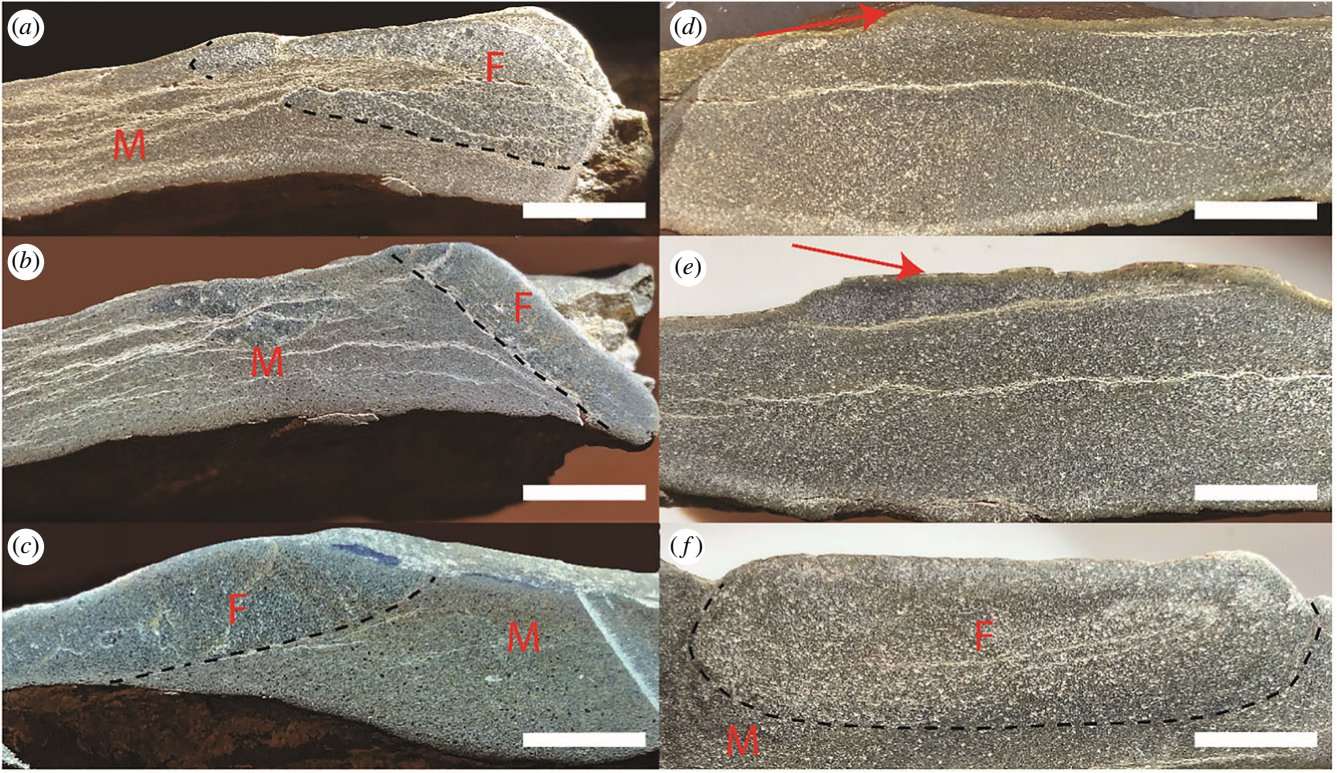
Polished cross sections of each fossil morphotype (one fossil sample each, three different sections). The ridged fossil (*a*–*c*) has a continuous interface; the ridgeless fossil (*d*–*f*) has a discontinuous interface. Black dashed lines indicate the interface between the fossil and the surrounding rock. Arrows in (*d*,*e*) point at the exposed surface of the fossil. Non-annotated polished sections are shown in electronic supplementary material, figure S1. F = fossil, M = matrix. Scale bars are 1 cm.

**Figure 3.**
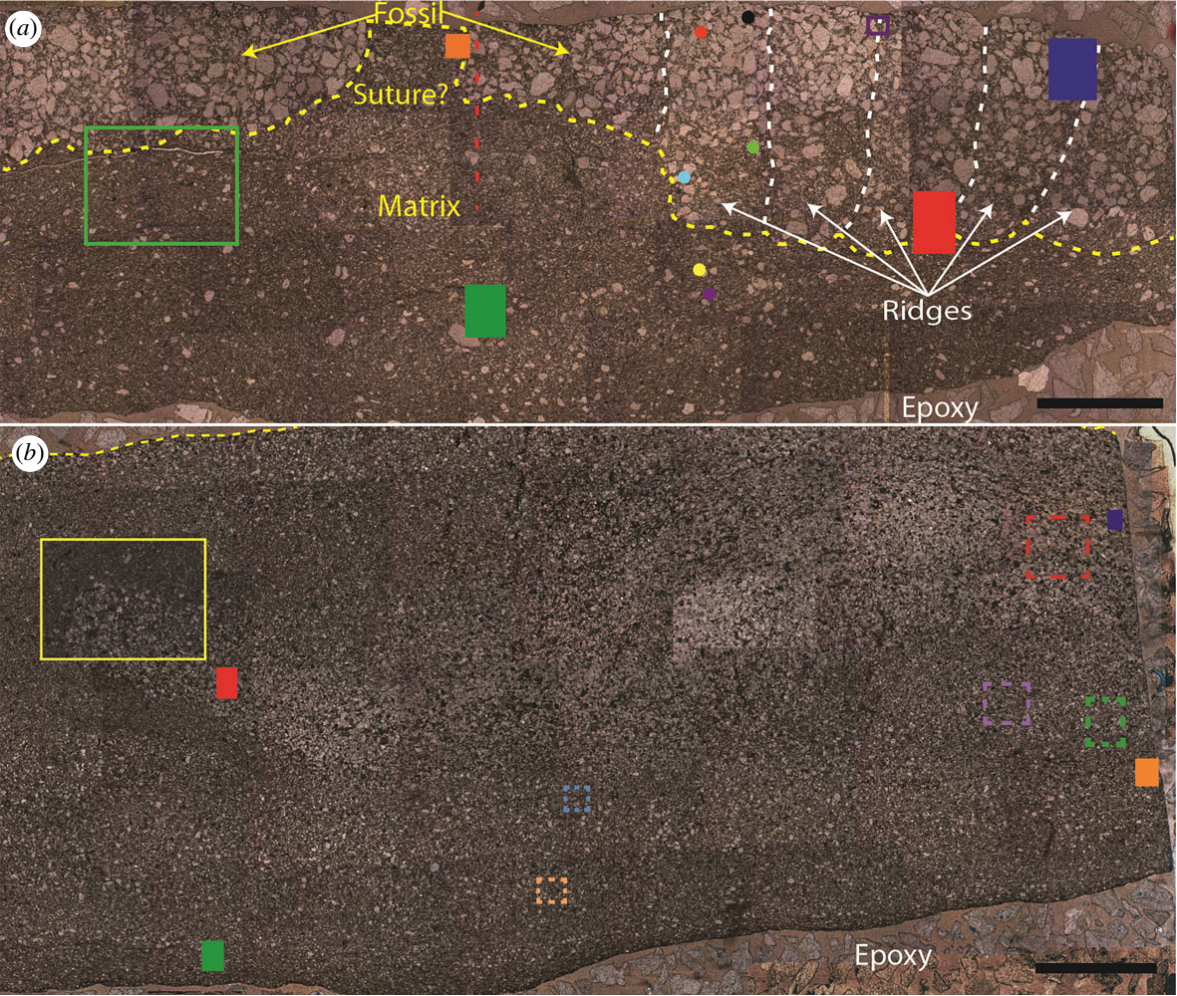
Photomosaics of the ridged (*a*) and ridgeless (*b*) erniettomorph fossils. (*a*) The ridged fossil contains medium to coarse angular quartz sand grains. The dashed yellow line follows a visible dark layer that traces the transition from coarse to very fine quartz grains at the lower interface between the fossil and the rock. The dashed red line marks the location of the XRD line scan. (*b*) The ridgeless erniettomorph fossil contains very fine to fine angular quartz sand grains and does not have a clearly defined lower or upper interface. The yellow dashed line indicates the exposed surface of the fossil as seen in figure 1*c*. Coloured shapes in (*a*) and (*b*) mark areas that were analysed by different techniques. Each shape in this figure corresponds to a technique. Unfilled, solid rectangles—reflectance and XPL petrography; unfilled, dashed rectangles—µXRD; filled rectangles—EDS; filled circles—Fe XANES. The results of analyses by these techniques are presented in the subsequent figures. Individual panels in these figures are outlined by colours that match the colours of the corresponding shape (e.g. EDS analyses of the area in the filled blue rectangle in (*a*) are outlined by a blue rectangle in figure 5*c*; petromicrograph of the area in the yellow rectangle in (*b*) is shown in figure 4*g*). Electronic supplementary material, figure S2, shows the same photomosaics without markings and annotations. Scale bars in both panels are 3 mm.

Three polished sections of each morphotype were cut and analysed to examine the interfaces between structures and the surrounding sediments (figure 2). In all polished sections of the ridged morphotype, the exposed fossil surface was elevated by 1–3 mm above the surrounding slab. A distinct lower boundary visually separated the 5–7 mm thick ridged fossil from the rest of the sample (figure 2*a–c*). Visual and microscopic examination of polished sections revealed that this boundary was defined by the grain size change and a dark mineral layer. By contrast, the interior boundary of the ridgeless fossil was discontinuous and only present in one of the polished sections (figure 2*d*–*f*). In areas where the exposed surface of the ridgeless fossil had a 1–3 mm higher relief compared to the surrounding rock, there was a visible dark boundary within the sample (figure 2*f*). Where this lower boundary was visible, the ridgeless fossil was 5–10 mm thick. Cracks within the polished sections ran parallel to the bedding outside of both fossil types and followed the interior boundary of the ridged fossil, indicating a plane of weakness within the rock. To compare the mineralogy and understand the origin of differences seen within the polished sections of the fossils, we analysed each fossil morphotype in thin section (figure 3).

### 3.2. Minerals in fossils and the surrounding sediment

Petrographic analyses of the entire vertical thin sections through fossils characterized the composition and distribution of minerals in the fossil and surrounding sediments. Quartz grains were abundant both within and outside of the ridged fossils, but the sizes of these grains differed. Medium to coarse, moderately sorted quartz sand grains were present within the fossils and very fine to fine sand grains were present outside the fossil (figure 3*a*). In the ridgeless fossil, the quartz grain size was very fine to fine and well sorted throughout the thin section (figure 3*b*). All quartz grains were sub-angular and did not show any evidence of quartz overgrowth that would indicate later precipitation of silica. The average size of the quartz grains was estimated from 45 randomly chosen areas from each thin section according to the method described above. Quartz grains in the ridged fossil were approximately 70% larger compared to those in the ridgeless fossil (*p*-value = 8.83 × 10^−8^). The spaces between quartz grains contained mostly clay minerals, with some feldspar, oxide and sulfide grains.

In the attempt to relate the abundances of quartz and clay minerals to the preservation of shape, we quantified the per cent area occupied by quartz in 45 randomly chosen fields of view from the ridged fossil. The clearly defined upper and lower boundaries therein enabled us to identify the following regions: within the fossil, at the interface and outside the fossil. ANOVA of these 45 fields of view revealed statistically significant differences (*p*-value ≤0.0042 for all three comparisons) among the mean per cent area occupied by quartz grains in all three regions. This area decreased from 53% within the fossil to 39% at the interface and then to 25% outside of the fossil. The ridgeless fossil lacked a clear interface, so areas within and outside could not be delineated with confidence. The average area occupied by quartz grains in this thin section was 33%. Thus, clay minerals occupied more spaces between the grains outside of the ridgeless fossil and within the structures that did not preserve ridges.

**Figure 4.**
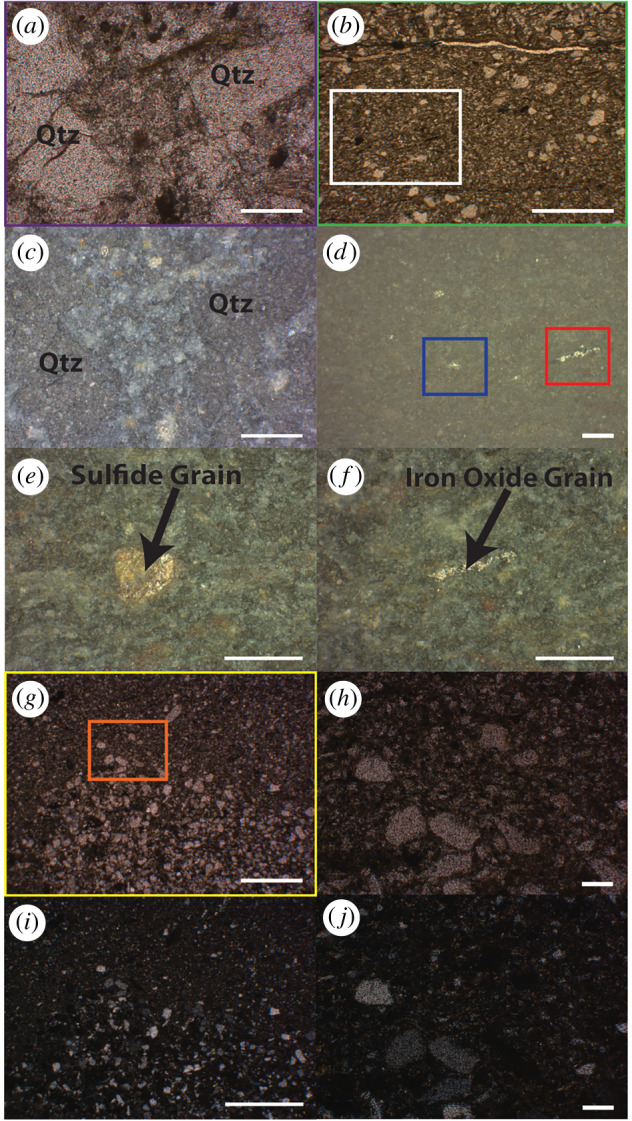
Photomicrographs of the ridged fossil (*a*–*f*) and the ridgeless fossil (*g*–*j*). Plane-polarized transmitted light (*a*,*b*,*g*,*h*), plane-polarized reflected light (*c*–*f*) and cross-polarized transmitted light images (*i*–*j*). The areas shown in (*a*,*b*,*g*) are identically coloured rectangles (purple, green, yellow) within the photomosaics in figure 3. (*e*,*f*) Sulfides and oxides in the blue and red boxes in (*d*), respectively, are sparsely distributed throughout the sample. (*i*,*j*) Clay minerals in cross-polarized transmitted light are more birefringent than surrounding quartz grains. Scale bar in (*b*) is 1 mm, all others are 100 µm.

**Figure 5.**
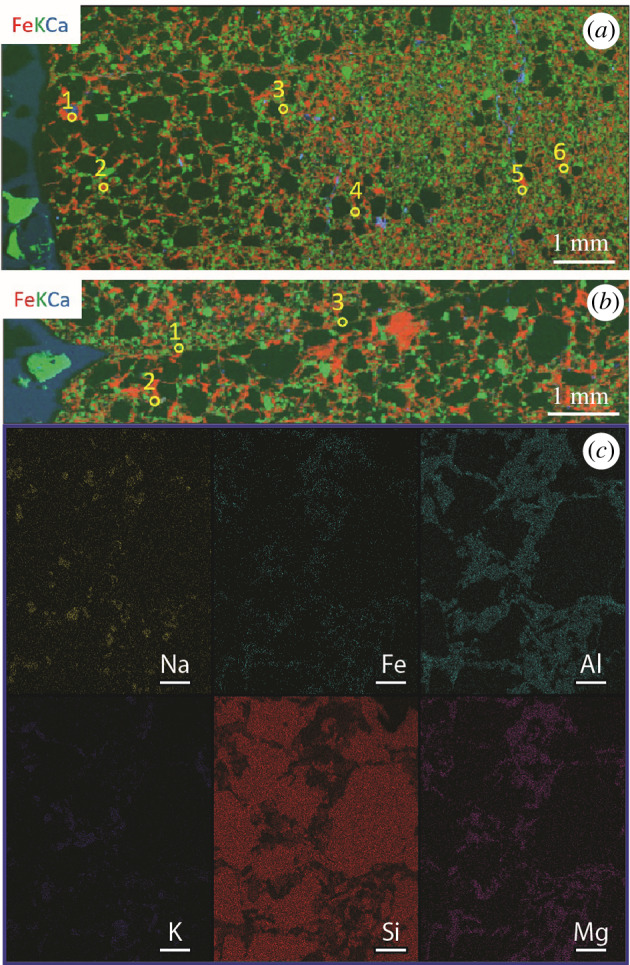
µXRF distribution maps of Fe (red), K (green) and Ca (blue) (*a*) inside and on (*b*) the surface of the ridged erniettomorph fossil. (*c*) Representative EDS map of the ridged fossil. All analysed areas show abundant aluminosilicate minerals between quartz grains (dark spots in *a*,*b*). Most analysed areas do not present evidence for oxide or sulfide mineral grains. The compositions of the clay minerals are consistent with chlorite (Mg-rich areas) and muscovite (K-rich areas) and are associated with the more abundant Fe. EDS data of the surrounding rock (electronic supplementary material, figure S3) and the ridgeless fossil (electronic supplementary material, figure S4) show similar patterns. The yellow, unfilled circles in (*a*,*b*) correspond to the filled circles in figure 3*a*. The area analysed in (*c*) is shown by the identically coloured blue rectangle in figure 3*a*. Scale bars in (*c*) are 100 µm.

**Figure 6.**
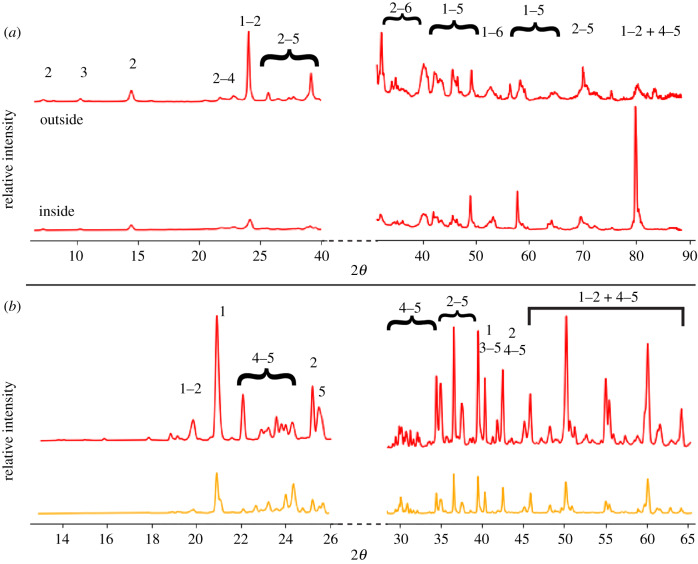
X-ray (*a*) and micro X-ray (*b*) diffraction patterns of the ridged and ridgeless fossil, respectively. Two representative spectra of each morphotype are plotted, but all spectra were similar and shown in electronic supplementary material, figure S5. (*a*) Spectrum generated from a line scan across the ridged fossil (location marked by the red-dashed line in the photomosaic in figure 3*a*). This line includes areas inside and outside of the fossil. (*b*) One-dimensional micro-XRD scans of different areas in the ridgeless fossil. These areas are marked by the unfilled rectangles of the same colour in the photomosaic in figure 3*b*. Phases identified by XRD on both cross sections: (1) quartz, (2) clinochlore, (3) muscovite, an assortment of (4) plagioclase and (5) potassium feldspars, and (6) calcite/ankerite.

Clay to silt sized grains filled all spaces between the quartz grains in both fossil types (figure 4). These grains had a higher order birefringence in cross-polarized light (XPL); some minerals also had a micaceous texture in plane-polarized light (PPL). All these minerals followed the boundaries of the quartz grains, never cross-cut other grains and exhibited rather uniform elemental composition. These characteristics are consistent with the properties of allochthonous clay minerals, suggesting that the precursor clay minerals were present in the original sediments. Potassium, iron and magnesium were the major cations in the clay minerals (figure 5), and XRD spectra of the fossils identified these minerals as iron-bearing clinochlore and muscovite (figure 6). The presence of chlorite and muscovite matched the observed abundances of magnesium and potassium, respectively. µXRF revealed Fe and K as major elements, and Ca occurred sporadically in some areas of the samples (figure 5*a*). Note that the Canberra detector used for this experiment cannot detect Mg, Al and Si. Fe K-edge XANES spectra of different spots in the ridged fossil identified Fe(II) as the prevalent valence state of iron. Chlorite was the main iron-bearing phase (electronic supplementary material, figure S6). Fe K-edge spectra of the surface of the ridged fossil suggested the presence of illite and kaolinite. These two clay minerals can form by various mechanisms that include the weathering of muscovite and chlorite, respectively, or the hydrolysis of feldspars (electronic supplementary material, figure S6). We consider the weathering of muscovite and chlorite to be the main source of illite and kaolinite, respectively, at the exposed fossil surfaces.

**Figure 7.**
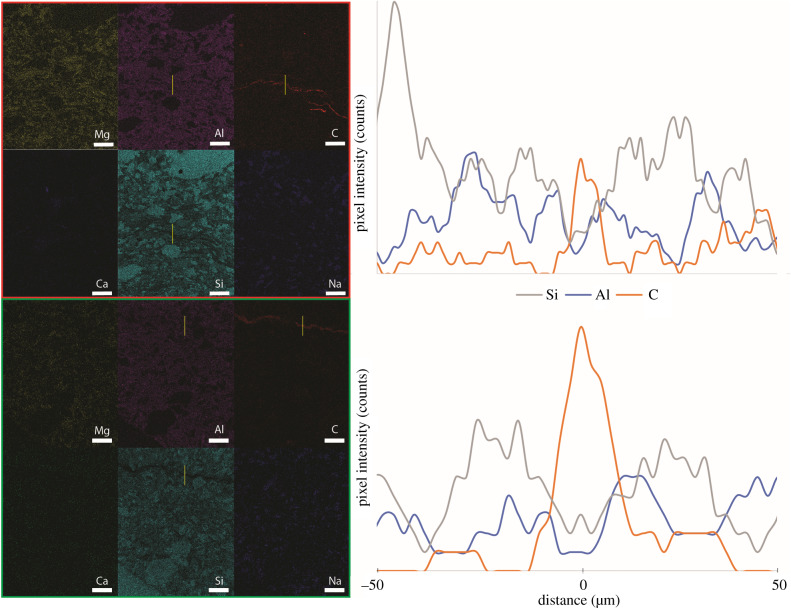
EDS maps of two carbon-rich regions in the thin section of the ridged fossil. The carbon enrichment at the fossil–rock interface coincides with the change of quartz grain size (red box, area shown by the filled red rectangle in figure 3*a*), but is also present below this interface (green box, area shown by filled green rectangle in figure 3*a*). The yellow lines in the EDS maps mark the locations of EDS line scans that span 50 µm on either side of the carbon laminae. Scale bar in all images is 100 µm.

Carbonates, sulfides, oxides and feldspars were present in both samples, but only as minor phases. Iron carbonate minerals, i.e. phases that can precipitate during early decay and diagenesis, were not detected in the areas of the samples investigated by Fe K-edge XANES. Moreover, carbonate minerals were not evident from the petrographic images of the samples in either PPL or XPL or from the elemental maps of the fossils. Nonetheless, the XRD spectra of the samples showed the presence of magnesian calcite and ankerite (figure 6*a*). Sulfides and oxides were noticeable as black mineral grains in transmitted light and as white-to-silver and yellow-to-gold coloured minerals in reflected light (figure 4). These minerals were sparsely distributed throughout the fossils and surrounding sediments and were not abundant enough to be detected by XRD. Thus, sulfate and iron were not reduced primarily at the interface between the soft-bodied organisms and the surrounding sediments or within the decaying organisms. All XRD spectra supported the presence of plagioclase and potassium feldspars throughout the samples (figure 6). Common in siliciclastic sediments, these minerals accounted for the observed enrichments of calcium and sodium in some grains mapped by EDS (figures 5 and 7).

### 3.3. Interface region/identification of fossil area

**Figure 8.**
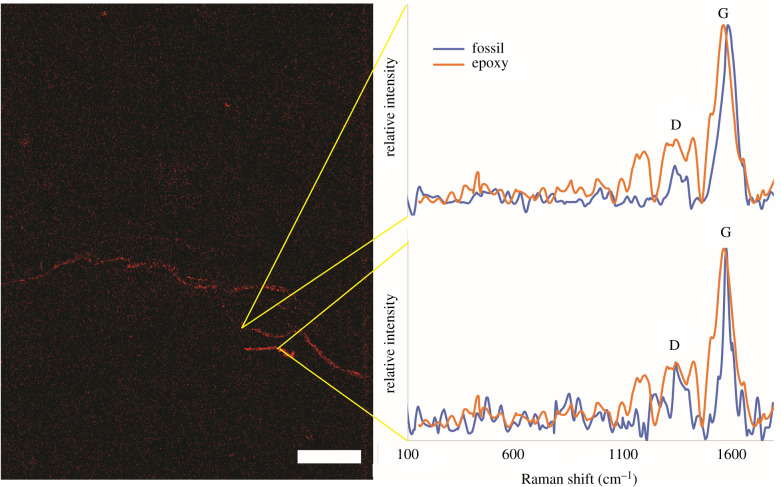
Raman spectroscopy of the carbon-rich areas of the ridged fossil and epoxy resin. The Raman spectra have the characteristic D- and G-band peaks of amorphous carbon. The G-band peak of the carbonaceous material in the fossil is shifted and much thinner, relative to that of epoxy resin. A band around 1200 cm^−1^ is present in the Raman spectrum of the epoxy resin and absent from the carbonaceous material at the fossil–rock interface. The same is true of the carbonaceous material in the ridgeless fossil (electronic supplementary material, figure S9). The same EDS carbon map can be seen in the top right panel of figure 7. Scale bar is 100 µm.

A continuous and visible interface within the ridged fossil separated the sample from the surrounding sediments (figure 2*a*–*c*). A dark and discontinuous interface was also observed in one cut through the flattened, ridgeless fossil (figure 2*f*). The visible boundary was defined partly by the differences in the grain size of quartz and in part by the presence of darker and finer grained clay material. The latter outlined the bottom ridges that were similar to those seen at the weathered surface of the sample (figure 3*a*). At this clay-rich interior boundary, the abundance of Al was similar to that inside and outside of the sample, but the abundance of Si was lower (figure 7), suggesting a different clay phase in the less than 20 µm thin boundary layer. EDS maps showed that a similarly thin layer of carbon was present in this region. This layer was not associated with a visible crack and the carbon signal intensity increases while the silicon signal intensity decreased (figure 7). The Raman spectrum collected from this carbon layer at the lower interface contained the D and G bands characteristic for thermally mature carbonaceous material (figure 8) [50]. This carbonaceous material differed from the epoxy resin and its thermal maturity suggested exposure to an approximately 500°C maximum temperature [51], which is well within the range expected by greenschist metamorphism. Based on these observations, the fossil boundary containing carbonaceous material and the Al-enriched clay phase likely formed during early decay and not during or after metamorphism or even later diagenetic fluid flow.

Other carbon-rich areas within the ridged fossil were less than 20 μm thin, farther away from the visible interface, and were not associated with any changes in quartz grain size (figure 7). The ridgeless fossil lacked equivalent petrographic distinctions, but a similar carbon-rich layer appeared at two different depths within the thin section (electronic supplementary material, figure S8). The depth of one matched the depth of the lower boundary of the ridged fossil, the other one was much deeper and closer to the bottom boundary of the entire thin section (electronic supplementary material, figure S8). These boundaries were not visible to the eye in the hand sample or thin section, in stark contrast to the boundary in the ridged fossil.

## 4. Discussion

The compositional and morphological differences between the two erniettomorphs suggest potential differences in taphonomic processes. Quartz grains were present in the precursor organisms of both fossils before burial. Two different interpretations of the lifestyle of precursor organisms are proposed for erniettomorphs from Namibia: some have suggested that these organisms were semi-infaunal, and used sediment as a ballast (e.g. [20,52–54]), while others have disputed this and suggested that erniettomorphs were infilled with sand post-mortem and pre-burial (e.g. [55]). In any case, sediment grains within the fossils from the WCF had to fill in the organisms before burial. Quartz grains present within the ridged WCF fossils are comparable in size to the fine-to-coarse sand grains seen within the Nama Group cast-and-mould fossils [33,56]. The larger quartz grain sizes found in the ridged WCF fossil suggest that it either lived in a more energetic area relative to the ridgeless fossil or that it experienced higher energy sedimentary processes such as bypass sedimentation very soon after death [57].

The abundant chlorite and muscovite within and outside of the fossils suggest that the precursor clay minerals were introduced into the original organisms before burial and were also present in sediments that buried these organisms. Because the WCF experienced lower greenschist facies metamorphism, the original clay minerals in and around the organisms were likely a mixture of kaolinite and smectite [58,59]. The presence of detrital clay minerals in both samples is consistent with the hypothesized roles of these minerals in the formation of cast-and-mould-style Ediacaran fossils [13,14,25,28]. For example, in experiments, authigenic and adsorbed clay minerals form veneers that are thinner than 100 µm around muscle tissues buried in pure kaolinite, whereas discontinuous greigite and iron oxides precipitate within tissues buried in sand or sand and illite mixtures [28]. Microbial degradation of soft tissues and the concurrent formation of clay veneers require sources of silica and potassium for the formation of authigenic clays and conditions conducive to, but without the rampant microbial reduction of iron [28]. These conditions are met because kaolinite can adsorb to organic compounds and reduce their decay by heterotrophic bacteria [25]. The microbial degradation under anoxic or suboxic conditions will also induce pH changes, dissolve kaolinite and increase the concentrations of silica [28] that can lead to the precipitation of authigenic clays. By contrast, the experimental decay of soft tissues in sand was associated with a larger pH decrease and the formation of greigite and iron oxide minerals [28]. These observations related the formation of early diagenetic minerals to the activity of heterotrophic microbes and pH changes induced by organic decay and demonstrated the dependence of this process on the composition and porosity of the surrounding sediment [28]. At present, it is unclear which features and early diagenetic minerals to expect in the mixtures of sand with kaolinite and smectite. Constraints on this are important for understanding taphonomic pathways that form Ediacaran cast-and-mould fossils that are found within clay-rich sandstones and siltstones but not within shale or quartz arenitic sandstone deposits.

We hypothesize that the type of sediment that buried these tissues may have controlled taphonomic processes in Ediacaran sandy and silty sediments. Continuous, thin layers of authigenic and detrital clay minerals are present at the interior boundary of the ridged fossils and also may have covered the now weathered kaolinite- and illite-rich surface. The observable associations of carbonaceous material with clay minerals (organominerals) at the fossil–sediment interface predate metamorphic activity and indicate that these associations formed during early organic decay and diagenesis. In three-dimensionally preserved erniettomorphs from the WCF, these minerals can preserve mm-scale diagnostic features of cast-and-mould fossils such as ridges. The presence of abundant clay minerals has been proposed as a predictive tool for the presence of Burgess Shale type preservation [31]. However, in contrast with the Ediacaran sandstones and siltstones that preserved erniettomorphs, the Burgess Shale-style preservation of reflective organic films occurred in fine clay matrixes that lacked larger quartz grains [6].

Pyritization and silicification have been suggested as fossilization mechanisms for cast-and-mould fossils at other localities (e.g. [1,11–13]). Pyritized body fossils of tubular organisms have been reported in other shale, siltstone and sandstone beds within the WCF [39,60,61] and the correlative Deep Spring Formation [40,61]. The WCF erniettomorphs occur in the same stratigraphic unit with the pyritized tubes, but do not present evidence for either pyritization or silicification [39]. In fact, the taphonomic windows that preserved tubular fossils in the WCF appear to have depended on the sediment porosity and composition. The pyritized tubular fossils in the WCF are restricted to approximately 3 m of green clays and siltstone [39,43], but cast-and-moulds of some of the same tubular fossils are found together with some erniettomorphs in siltstone and sandstone at other stratigraphic intervals of the WCF [39]. If pyrite or other iron sulfide minerals had originally precipitated on the decaying walls of sand-filled organic sacs of the erniettomorphs, these minerals or their oxidized weathering products would have been concentrated at the fossil walls [11,12,62]. Instead, sulfides and oxides were sparsely present throughout both samples and the surrounding sedimentary matrix. Thus, it is unlikely that microbial sulfate and iron reduction were confined to the organic walls only, even though they preserved the walls of pyritized tubular fossils in nearby green clays and siltstones [39,40]. The lack of microbial structures within the fossiliferous sandstone beds [39] and in hand samples also does not provide evidence for sealing organic layers where sulfate- and iron-reducing microbes were particularly active [11,12,62]. Overall, the taphonomic windows that preserved fossils in the WCF appear to have depended on the sedimentation rate, sediment porosity and composition. Among these taphonomic windows, the more porous and quartz-rich sediments favoured the preservation of erniettomorphs.

The results presented here differ from previous taphonomic characterizations of erniettomorphs and other Ediacaran biota and suggest that mechanisms that preserved different Ediacaran cast-and-mould fossils were a complex function of the type of and relative amounts of minerals, grain sizes and organic matter. By contrast with the WCF erniettomorphs, three-dimensionally preserved erniettomorph fossils from the Nama Group are found within quartz-rich sedimentary strata [17,33,53,63,64]. Clay minerals are not as abundant in these specimens, but many of the reported Nama Group fossils preserve fine features at a similar scale to the millimetre-scale ridges of the ridged WCF fossil. However, the WCF fossils analysed here appear to be flattened more than the Nama Group fossils [17,33,53,63,64]. The fossiliferous Ediacara Member, the type section for the Ediacaran biota, is composed of quartz-feldspar arenites with sparse clay minerals [1,38] and contains numerous cast-and-mould fossils with mm-scale features (e.g. [1,38]). In general, the clay-rich sandstones that preserve the fossils analysed in this study are not a lithology typically associated with other Ediacaran cast-and-mould deposits.

**Figure 9.**
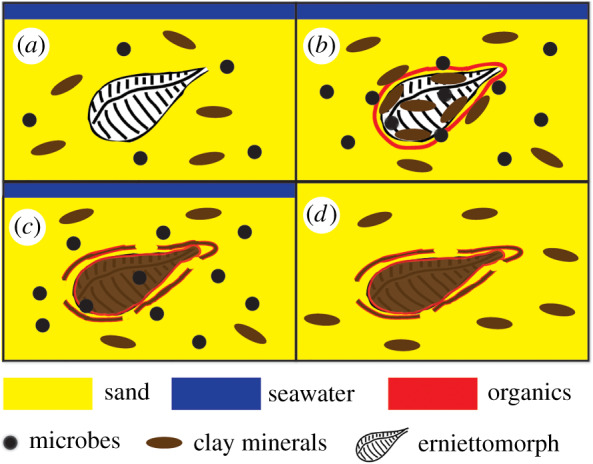
Cartoon model of early fossilization of the ridged erniettomorph fossils. (*a*) Organisms that were filled by quartz-rich sediments were transported and buried in clay-rich sediments. (*b*) Microbially mediated decay started shortly after death and burial. Clay minerals adsorbed onto the surfaces of organic material. (*c*) Over time, a Si-poor and Al-rich clay mineral precipitated and bound some organic compounds that were present at the surface of the decaying organism. Clay minerals also bound organic compounds that diffused away from the organisms. Some flattening occurred. (*d*) Additional flattening occurred after additional burial and later metamorphism, but three-dimensional structures and millimetre-scale ridges were retained due to the presence of quartz-rich infill.

The association of clay minerals and Ediacaran soft-bodied fossils has been described before [13,15,34,52], but the processes controlling the formation of two-dimensional versus three-dimensional fossils and the preservation of mm-scale features and potential suture lines (e.g. [1,13,33,39,52]) have not been fully explored. Clay minerals are known to inhibit or limit the microbial activity [25,28] and help preserve organic compounds and soft-bodied organisms (e.g. [6,65]). With this in mind, if the ridgeless structures are indeed taphomorphs of formerly ridged erniettomorphs, their poor preservation is surprising, because their precursor organisms contained *more* clay before burial compared to the ridged organisms and were buried in sediments that contained abundant clay minerals. A recent study reported the flattening of muscles buried in kaolinite [28]. The same flattening process may have contributed to the disappearance of mm-scale ridges. Hence, the lower abundances of clay minerals in the interiors of organisms filled with the coarser quartz grains may actually account for the lesser extent of flattening and the better preservation of millimetre-scale ridges during early decay, where these fossils were preserved at all. Our proposed mechanism is summarized in figure 9. These interpretations are consistent with the recent descriptions of *Ernietta* from the Nama Group. For example, Elliot *et al*. [52] and Ivantsov *et al*. [53] report the more common three-dimensional preservation of the proximal structures of erniettomorphs from the Nama Group compared to the distal structures. They interpret the former as residing within sediments and likely filled with sediments before transport and the latter as present above the sediment–water interface and fluid-filled before burial. Ivantsov *et al*. [53] also argue that the only preserved distal structures contained sediment infill from during both transport and subsequent burial. This interpretation requires a semi-endobenthic lifestyle for erniettomorphs (e.g. [18] and references therein), in contrast with the inferred epibenthic lifestyle of most other Ediacaran organisms (e.g. [18] and references therein).

Additional mechanisms may have given rise to the differences between the ridged and ridgeless fossils. For example, Retallack [66] interprets ribbed fossils as external casts and smooth fossils as internal casts. This model would indicate that the ridged and ridgeless fossils are external and internal casts, respectively, of the same organisms. If so, the exceptional preservation of external casts and their mm-scale ridges in the WCF was associated with the formation of continuous organomineral layers around organisms that were filled by coarse grained quartz. Discontinuous and less visually prominent layers are associated with the less well-preserved internal casts, but the presence of carbonaceous laminae in both the ridged and the ridgeless fossils strengthens the case for the interpretation of the two morphologies as taphomorphs. Taphonomy experiments that elucidate the role of different clay minerals and sand in different stages of fossilization can help develop a better understanding of the conditions conducive to the preservation of different tissue types and diagnostic characters.

## 5. Conclusion

Abundant quartz grains and clay minerals in erniettomorph fossils from the lower WCF may have had different roles in the cast-and-mould-style preservation. Angular to sub-angular quartz grains and the clay minerals, kaolinite and smectite, filled the interiors of the erniettomorph organisms and were present in the enclosing sediments. Sand grains maintained the macroscopic three-dimensional morphology of the organisms during decay. Evidence for similar contributions by the sparsely distributed iron oxides and sulfides produced by the microbial reduction of sulfate and iron is lacking. Clay minerals adsorbed onto the surfaces of organisms during the early stages of decay, delaying the decay and preserving carbonaceous matter at thin, but visible organomineral interfaces that separated the decaying organisms and the surrounding sediment. Microbial activity under reducing conditions also transformed some original clays into a thin layer of authigenic clays at the sediment–fossil interfaces. These boundaries may have contributed to the differential weathering of the fossils from the surrounding sandstone and siltstone. The later greenschist metamorphism likely altered kaolinite to chlorite, smectite to muscovite and authigenic clays to a currently uncharacterized phase, and led to the partial graphitization of the carbonaceous material within the sediments and at the fossil–sediment interfaces. Our study indicates that clay minerals, where present, can help preserve organic matter during cast-and-mould-style fossilization. Although this process is associated with the vertical flattening, abundant quartz grains in Ediacaran sandstone deposits enabled the preservation of three-dimensional structures and mm-scale diagnostic features in cast-and-mould fossils.

## Supplementary Material

Supplementary Figures and Table

## Data accessibility

Data used within this study are available from the Dryad Digital Repository: https://doi.org/10.5061/dryad.qz612jm9r [67].
