# Supplementary material for: Preservation of erniettomorph fossils in clay-rich siliciclastic deposits from the Ediacaran Wood Canyon Formation, Nevada
Source: Interface Focus. 2020 Jun 12;10(4):20200012. doi: 10.1098/rsfs.2020.0012 (PMC7333903; doi:10.1098/rsfs.2020.0012)
Supplement: Supplementary Figures and Table [file rsfs20200012supp1.docx]

**Supplementary material: ‘Preservation of erniettomorph fossils in clay-rich siliciclastic deposits from the Ediacaran Wood Canyon Formation, Nevada’**

**Supplemental Figures**

**
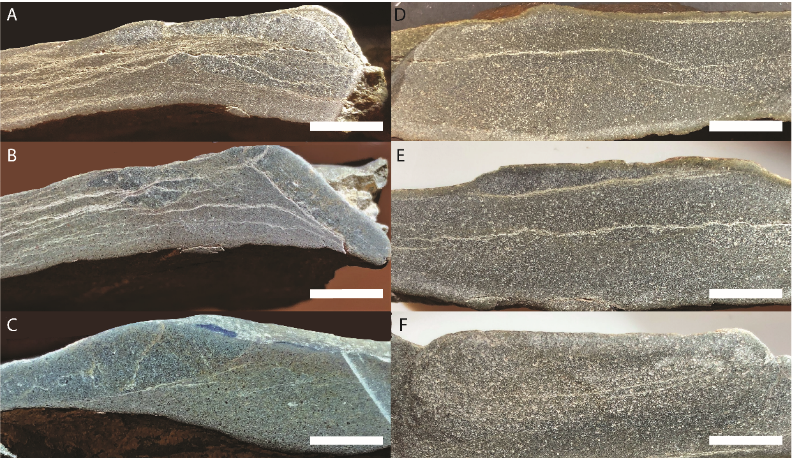
**

**Supplemental Figure 1. Polished cross sections through the ridged fossil (A-C) and ridgeless fossil (D-F). Annotated polished sections are shown in Figure 2. Scale bars are 1 cm.**

**
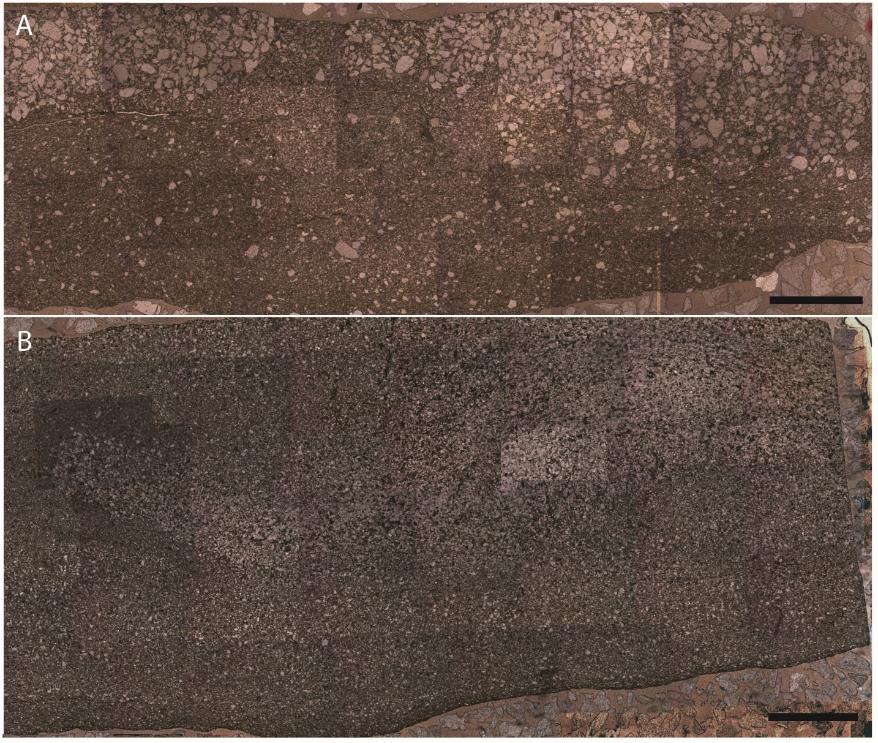
**

**Supplemental Figure 2. Non-annotated photomosaics of the ridged (A) and ridgeless (B) erniettomorph fossil thin sections. Annotated photomosaics are shown in Figure 3. Scale bars are 3 mm.**

**
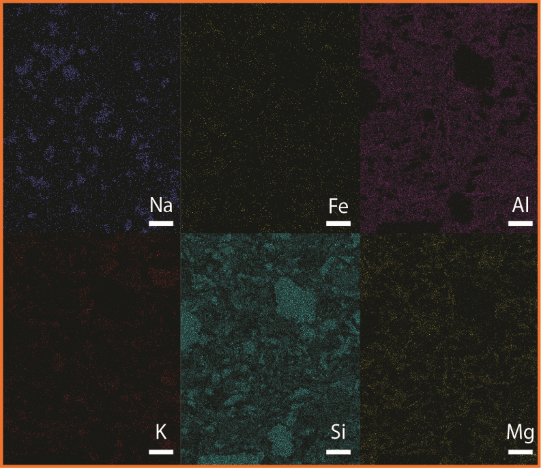
**

**Supplemental Figure 3. Representative EDS map of the rock outside of the ridged fossil. All analysed areas show abundant aluminosilicate minerals between quartz grains and most analysed areas present no evidence for oxide or sulphide mineral grains. The compositions of the clay minerals are consistent with chlorite (Mg rich areas) and muscovite (K rich areas) and are associated with increased Fe. Similar patterns are seen in the EDS data of the ridged fossil (Figure 5). The colour of the box (orange) ­matches the colour of the rectangles outlining the areas shown in the photomosaic in Figure 3a. Scale bars are 100 µm.**

**
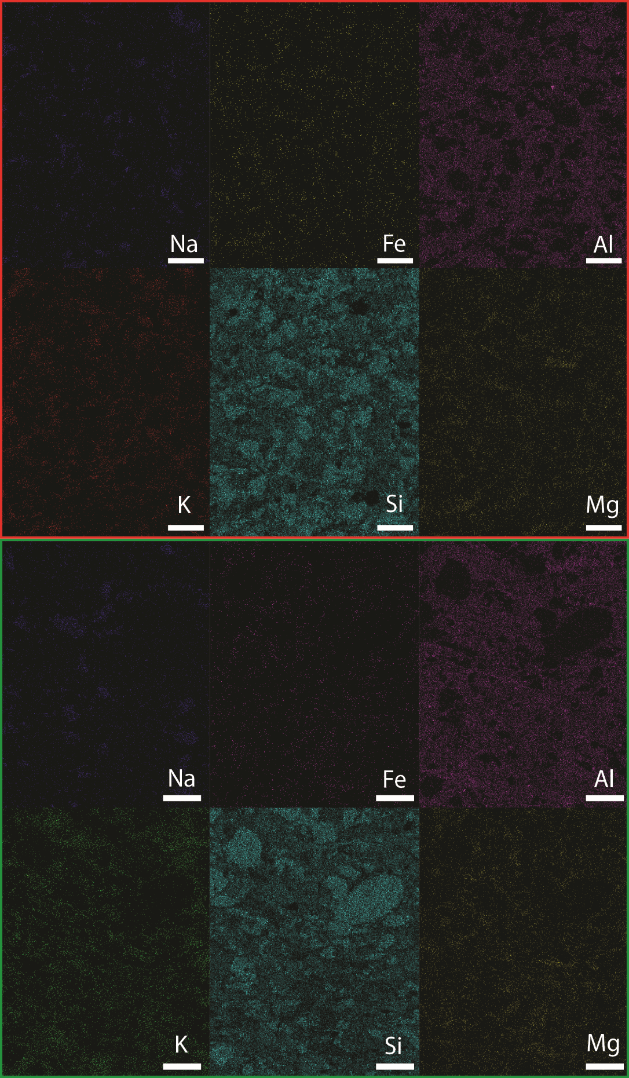
**

**Supplemental Figure 4. Representative EDS maps of the ridgeless fossil. Similar patterns are seen within the ridged fossil EDS data (Figure 5). The colour of the boxes (red or green) matches the colour of the rectangles outlining the areas shown in the photomosaic in Figure 3b. Scale bars are 100 µm.**

**
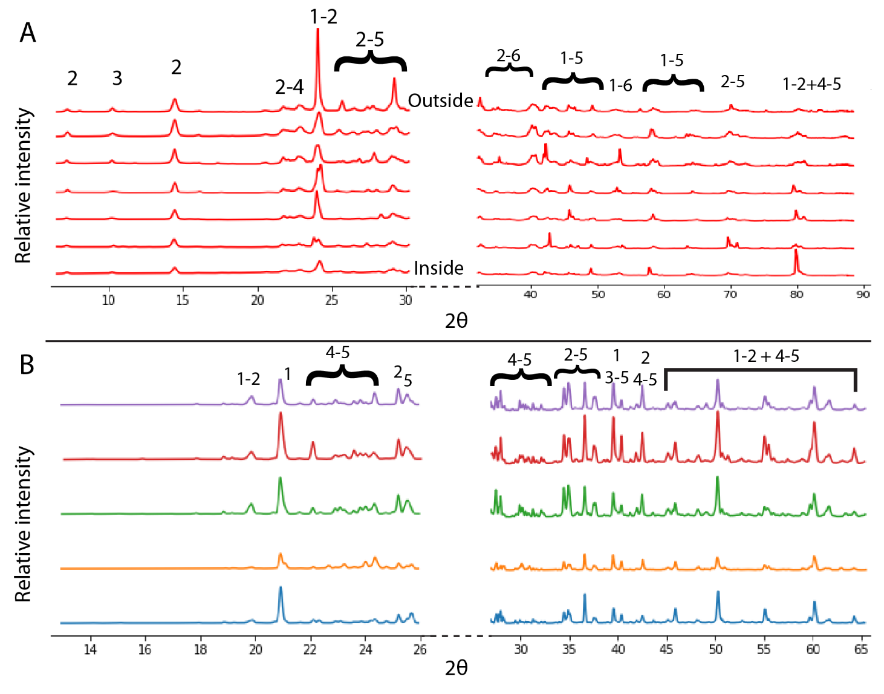
**

**Supplemental Figure 5. X-ray (A) and micro X-ray (B) diffraction patterns of the ridged and ridgeless fossil respectively. A) Spectra generated from a line scan across the ridged fossil, shown as the red-dashed line in the photomosaic in Figure 3a. These are labelled as whether being inside the fossil, or outside of the fossil. B) 1-D micro-XRD scans of different areas in the ridgeless fossil, shown as unfilled rectangles of the same colour in the photomosaic in Figure 3b Phases identified by XRD on both cross-sections are similar: (1) quartz, (2) clinochlore, (3) muscovite, an assortment of (4) plagioclase and (5) potassium feldspars and (6) calcite/ankerite.**

**
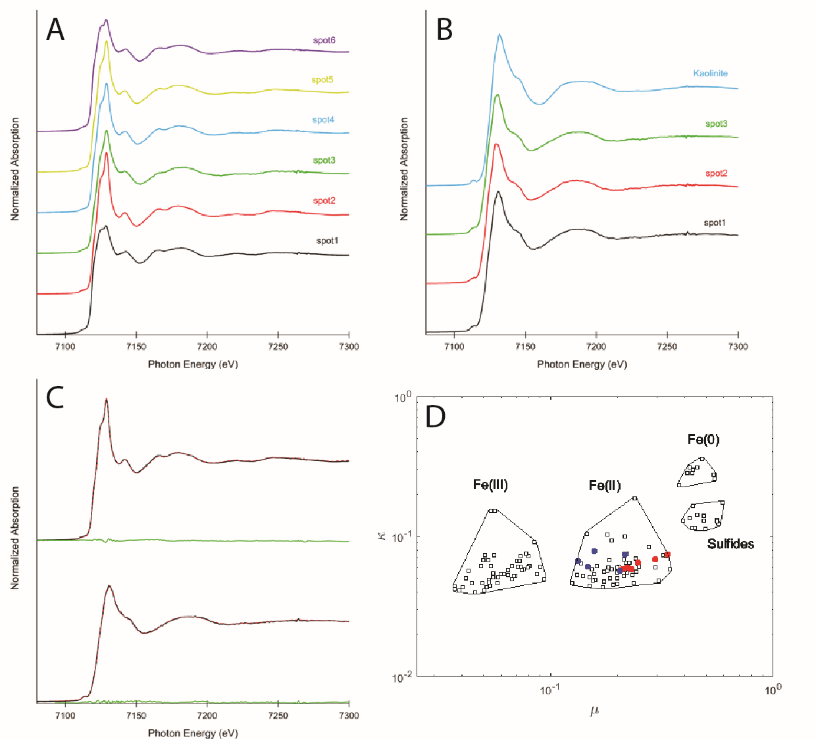
**

**Supplementary Figure 6. A-B) Fe K-edge XANES spectra collected from the regions circled in yellow in Figure 5a-b. C) Example of LSQ LC fittings (fits in red, residuals in green) of spectra collected on the inside (spot2) and surface (spot1), see table S1 for details. D) Fe valence scatter plot generated from the ridged erniettomorph fossil spectra (fossil data points in color, Fe standards in black open squares) showing Fe(II) in the samples as confirmed by LSQ fittings. The color of the spectra in panel A matches the colored dots in Fig. 3a. The points from the surface best matched a mixture of clays (see table S1) mostly illite and kaolinite, i.e., the respective weathering products of muscovite and chlorite.**

**
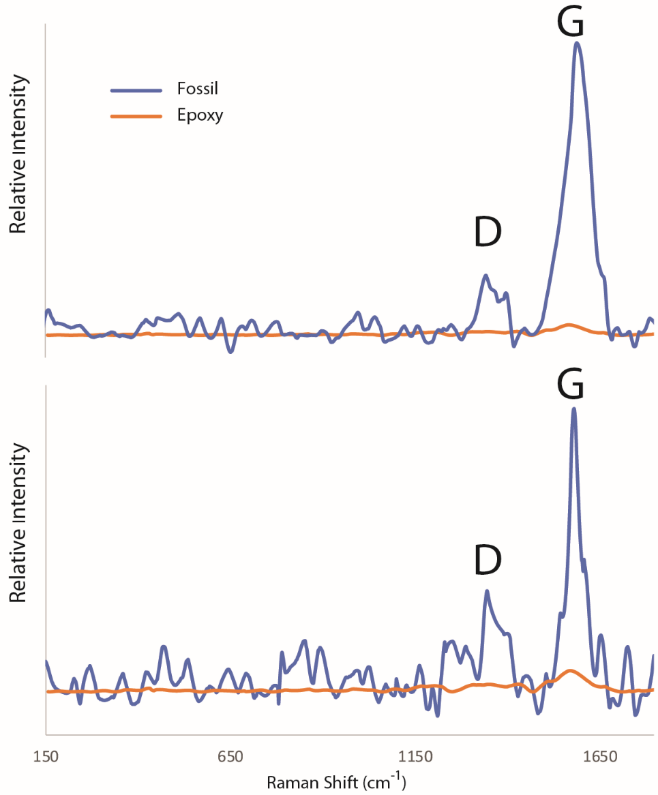
**

**Supplemental Figure 7. Raman spectroscopy of the carbon rich areas of the ridged fossil as shown in Figure 8. The spectra are shown as the ratios of the baseline-subtracted Raman spectra divided by the maximum value found in the fluorescent region, the region past 1700 cm^-1^ (50). The carbonaceous material in the ridged fossil is less fluorescent and thus more thermally mature than the epoxy resin (50). A similar trend is seen for the ridgeless fossil shown in Supplemental Figure 10.**

**
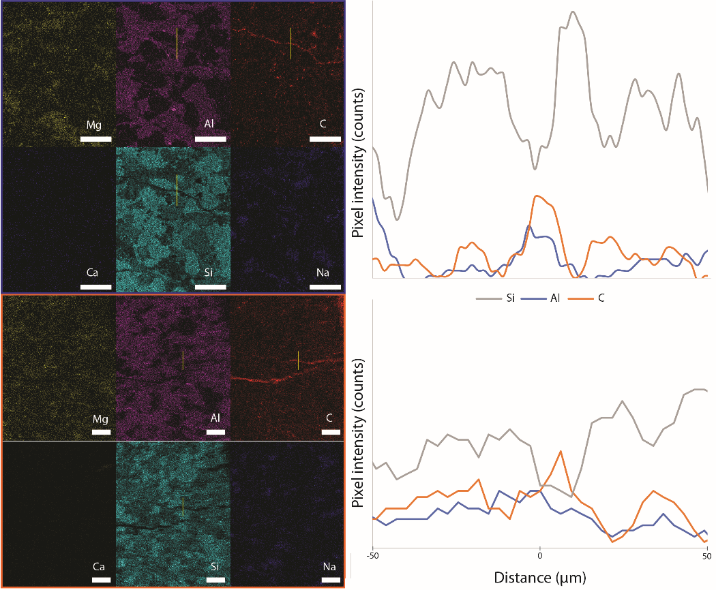
**

**Supplemental Figure 8. EDS maps of the carbon rich regions of the ridgeless fossil. The distribution of carbon matched what is seen in the ridged fossil (Figure 7), however, the places where they occur are not the same. The colour of the boxes (blue or orange) matches the colour of the rectangles outlining the areas shown in the photomosaic in Figure 3b. The intensity line profiles are across the yellow line on the EDS maps, which shows 50 µm on either side of the carbon lamina. These show that the intensity of Si decreases and the Al intensity remains the same where the C intensities increase. Scale bar in all images is 100 µm.**

**
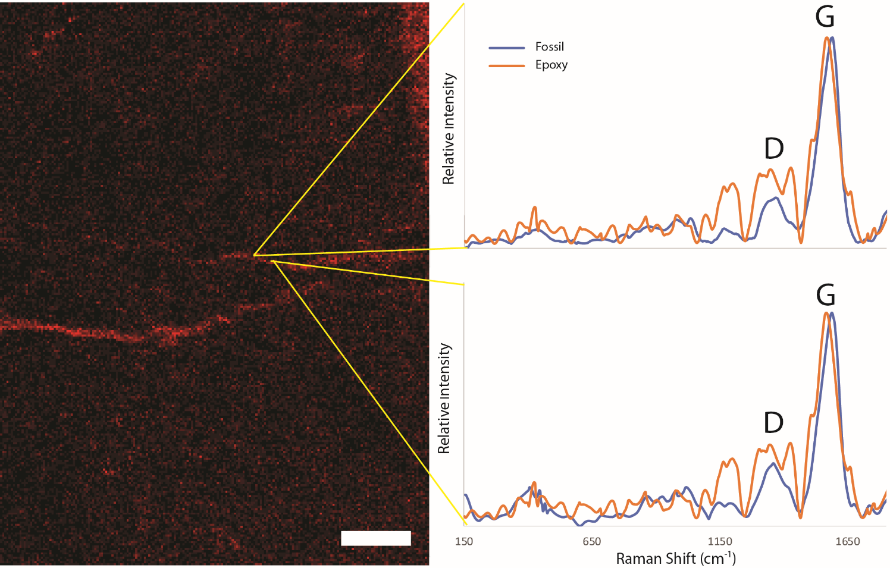
**

**Supplemental Figure 9. Raman spectroscopy of the carbon rich areas of the ridgeless fossil and epoxy resin. The Raman spectra have the characteristic D and G band peaks of amorphous carbon. The G-band peak of the carbonaceous material in the fossil is shifted and much thinner, the band around 1200 cm^-1^ seen in epoxy resin is missing from the carbonaceous material at the boundaries. Similar trends are seen in the ridged fossil (Figure 8). EDS image is the same as the panel labelled ‘C’ in the top boxed region of Supplemental Figure 8. Scale bar is 100 µm.**

**
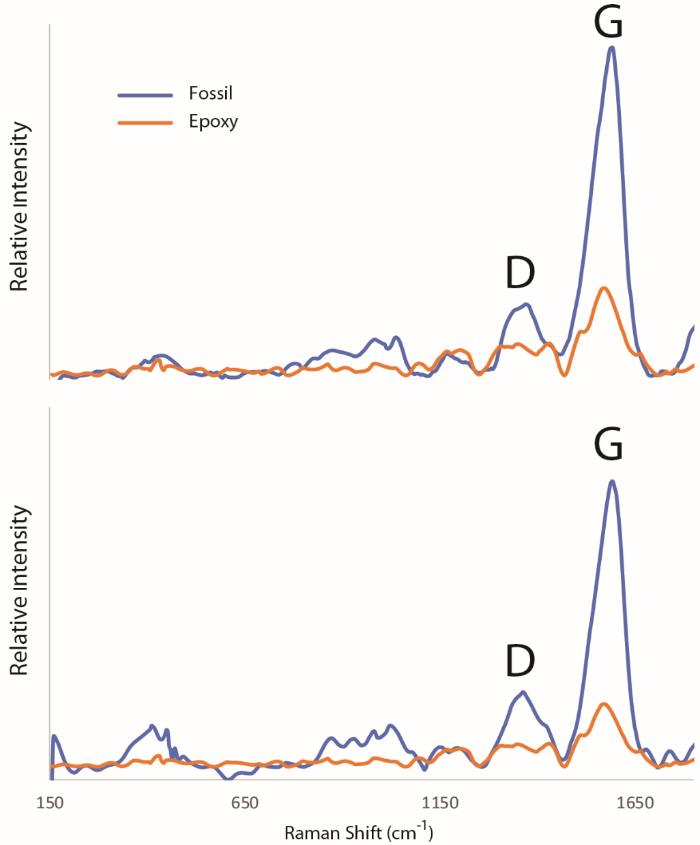
**

**Supplemental Figure 10. Raman spectroscopy of the carbon rich areas of the ridgeless fossil as shown in supplemental Figure 9. The spectra are shown as the ratios of the baseline-subtracted Raman spectra divided by the maximum value found in the fluorescent region, the region past 1700 cm^-1^ (50). The carbonaceous material in the ridged fossil is less fluorescent and thus more thermally mature than the epoxy resin (50). A similar trend is seen for the ridged fossil shown in supplementary Figure 7.**

**TABLE S1. Results of LSQ linear combination fittings of Fe K-edge XANES data.**

**Sample XANES spot# Minerals identified Amounts (%) sum-sq. sum**

**Erniettomorph**

**Inside** **1** **- - - -**

**2** Chlorite(Ripidolite)_CCa2 57 7.50E-5 103

Chlorite_R2139 46

**3** Kaolinite 24 8.21E-5 100

Chlorite_r2137 70

Pyrrhotite 6

**4** chlorite_r2139 73 9.34E-5 100

FeSi 8

Hypersthene 19

**5** Arsenopyrite 8 1.06E-4 100

Chlorite_R2139 92

**6 - - -**

**Erniettomorph**

**Surface** **1** brucite 21 5.72E-5 100

illite_imt-1 clay 52

kaolinite_kga-1b 27

**2** brucite 26 7.03E-5 101

chlorite(ripidolite)_cca2 25

illite_smectite_mixed_iscz-1 50

**3** chlorite_r2139 29 8.04E-5 100

Fe3Si 7

kaolinite_kga-1b 64

*Notes: -: no good fit.. The best LSQ fit was obtained by minimizing the normalized sum-squares residuals: NSS = 100 × {∑(µexp – µfit )^2^/ ∑ (µexp)^2^} in the 7010-7410 eV range, where µ is the normalized absorbance. Errors on fits are +/- 10%. The Fe XAS database does not include a muscovite standard.*
